# Growing a positive culture in an ICU antimicrobial stewardship program

**DOI:** 10.1186/cc11109

**Published:** 2012-03-20

**Authors:** K Walker, J Litynsky, J Powis

**Affiliations:** 1 Toronto East General Hospital, Toronto, Canada

## Introduction

A 3-month pilot antimicrobial stewardship program (ASP) was initiated in a 490-bed urban community hospital medical/surgical ICU. The ASP continued post pilot. ASP goals are to optimize/reduce antimicrobial (AM) usage, improve clinical outcomes and reduce nosocomial *Clostridium difficile *rates [1,2].

## Methods

The pilot had one pharmacist (Ph) providing ICU clinical service and one AMPh, both working as ICUPhs. The AMPh collected standardized data on patients and were reviewed with the ID physician; then the AMPh and ID physician discussed with the ICU care team for optimal AM use. Post pilot, the ICUPh assumed the AM stewardship role. The ASP reduced from 5 to 3 days/week. Data collection included the ASP time required and interventions. The same metrics were collected pre/post pilot.

## Results

The ASP total patient recommendations/100 patient-days were 5-day mean 9.3, 3-day mean 13.5 (*P *= 0.030) with an increased ICU physician acceptance (5 days = 95.9%, 3 days = 99.7%). Statistically significant was an increase in recommendations to broaden therapy (Table 1) and nonstatistically significant was a reduction in recommendations to de-escalate therapy (5-day mean 1.4 recommendations/100 patient-days, 3-day mean 1.2 recommendations/100 patient-days; *P *= 0.601). Also, there was an increase in recommendations for duration optimization (5-day mean 4.0 recommendations/100 patient-days, 3-day mean 6.0 recommendations/100 patient-days; *P *= 0.055) and discontinue AMs (5-day mean 2.7 recommendations/100 patient-days; 3-day mean 3.7 recommendations/100 patient-days; *P *= 0.181). The ASP mean time required (minutes/month) was reduced (5 days 864, 3 days 771; *P *= 0.267).

**Table 1:** 

Recommendations/100 patient-days	ASP days		
		
	5-day mean	3-day mean	*P *value
Broaden	0.4	1.6	0.003

## Conclusion

ASP reduction from 5 to 3 days/week was successful. Necessary skills were developed by the ICUPh. ASP reduction increased recommendations for discontinuation and prospective duration optimization goals of AMs. A reduction in recommendations to deescalate therapy and an increase in broadening therapy may reflect an increased acceptance goals. The 3-day ASP also demonstrated an increase in total recommendations/100 patient-days and a reduction in the total time required which enhanced use of resources, both financial and human.
